# Tetraspanin Family Member, CD82, Regulates Expression of EZH2 via Inactivation of p38 MAPK Signaling in Leukemia Cells

**DOI:** 10.1371/journal.pone.0125017

**Published:** 2015-05-08

**Authors:** Chie Nishioka, Takayuki Ikezoe, Jing Yang, Akihito Yokoyama

**Affiliations:** 1 Department of hematology and Respiratory Medicine immunology, Kochi Medical School, Kochi University, Nankoku, Kochi 783–8505, Japan; 2 Department of immunology, Kochi Medical School, Kochi University, Nankoku, Kochi 783–8505, Japan; Fujita Health University, School of Medicine., JAPAN

## Abstract

**Purpose:**

We recently found that the tetraspanin family member, CD82, which is aberrantly expressed in chemotherapy-resistant CD34^+^/CD38^−^ acute myelogenous leukemia (AML) cells, negatively regulates matrix metalloproteinase 9, and plays an important role in enabling CD34^+^/CD38^−^ AML cells to adhere to the bone marrow microenvironment. This study explored novel functions of CD82 that contribute to AML progression.

**Materials and Methods:**

We employed microarray analysis comparing the gene expression profiles between CD34^+^/CD38^−^ AML cells transduced with CD82 shRNA and CD34^+^/CD38^−^ AML cells transduced with control shRNA. Real-time RT-PCR and western blot analysis were performed to examine the effect of CD82 knockdown on the expression of the polycomb group member, enhancer of zeste homolog 2 (EZH2), in leukemia cells. A chromatin immunoprecipitation assay was performed to examine the effect of CD82 expression on the amount of EZH2 bound to the promoter regions of tumor suppressor genes in leukemia cells. We also utilized methylation-specific PCR to examine whether CD82 expression influences the methylation status of the tumor suppressor gene promoter regions in leukemia cells.

**Results:**

Microarray analysis revealed that levels of EZH2 decreased after shRNA-mediated depletion of CD82 in CD34^+^/CD38^−^ AML cells. Moreover, the antibody-mediated blockade of CD82 in leukemia cells lowered EZH2 expression via activation of p38 MAPK signaling, decreased the amount of EZH2 bound to the promoter regions of the tumor suppressor genes, and inhibited histone H3 lysine 27 trimethylation in these promoter regions, resulting in upregulation of the tumor suppressors at both the mRNA and protein levels.

## Introduction

Acute myelogenous leukemia (AML) originates from hematopoietic stem/progenitor cells and is maintained by a subset of leukemia stem cells (LSCs), which are assumed to be enriched for the CD34^+^/CD38^−^ fraction [1–3]. We have recently shown that CD34^+^/CD38^-^ AML cells are in a dormant state and are more refractory to the anti-leukemia agent, cytarabine, than mature CD34^+^/CD38^+^ AML cells [4]. Therefore, a better understanding of the molecular mechanisms that allow CD34^+^/CD38^-^ AML cells to escape chemotherapy appears necessary to improve the prognosis for individuals with AML.

The mitogen-activated protein kinase p38 (p38 MAPK) regulates the cell proliferation, differentiation, apoptosis, and senescence [5–8]. It is noteworthy that p38 is less phosphorylated in CD34^+^/CD38^−^ AML cells than in normal hematopoietic stem cells (HSCs) and H_2_O_2_-induced senescent HSCs [9].

Polycomb group proteins are multifaceted regulators of both normal and cancer stem cells, and are involved in the regulation of stem cell self-renewal and fate determination [10]. The enhancer of zeste homolog 2 (EZH2), a member of the histone methyltransferase family, catalyzes the trimethylation of histone H3 at lysine 27 (H3K27me3) and is located in 7q36.1 [11,12]. EZH2 mutations were found in 7% of follicular lymphomas and 22% of diffuse large cell B-cell lymphomas [13]. These mutations cause reduction of p16 expression and are recognized as gain of function mutations [14]. In myeloid neoplasms, EZH2 mutations were identified in 10–13% of poor-prognosis myelodysplastic syndromes-myeloproliferative neoplasms, 6% of myelodysplastic syndromes, 6% of chronic myelomonocytic leukemia, and 1.7% of AML [13, 15–17]. EZH2 mutations in myeloid neoplasia were characterized by decreased H3K27me3 and increased chromatin relaxation at specific gene loci accompanied by higher transcriptional activity [18].

We previously found that long-term exposure of leukemia cells to imatinib induces expression of DNA methyltransferase 3A (DNMT3A) and EZH2, which then interact with one another and suppress the expression of phosphatase and tensin homolog deleted on chromosome ten (PTEN) in leukemia cells [19]. In addition, we recently found that hypermethylation of *PTEN* is associated with downregulation of *PTEN* transcription in imatinib-resistant leukemia cells isolated from individuals with chronic eosinophilic leukemia, chronic myeloid leukemia (CML), and Philadelphia positive acute lymphoblastic leukemia (Ph^+^ ALL) [20].

The *p16* gene, also known as cyclin dependent kinase inhibitor 2 (*CDKN2A*) and cyclin-dependent kinase 4 inhibitor (*CDK4I*) [21], plays an important role as a tumor suppressor gene. De novo methylation of CpG sequences in the *p16* promoter occurs in approximately 30% of colorectal tumors [22–24] and is considered to be important in the pathogenesis of this particular malignancy. Hypermethylation around the promoter region of *p16* has also been found in AML. Interestingly, the DNMT inhibitor, azacytidine, is associated with hypomethylation of *p16*, and is known to suppress proliferation of leukemic cells [25].

CD82 is a member of the tetraspanin family, many of which are implicated in the regulation of cell motility, morphology, fusion, signaling, fertilization, and differentiation [26]. CD82 was originally identified as a suppressor of metastatic spread in a rat prostate cancer model [27–29]. Recent studies showed that CD34^+^/CD38^−^ cells isolated from individuals with AML displayed higher CD82 expression levels than CD34^+^/CD38^−^ cells from healthy donors. Notably, CD82 negatively regulated matrix metalloproteinase 9 (MMP9) and modulated adhesion to bone marrow (BM) and survival of AML cells via the STAT5/IL-10 signal pathway [30, 31]

Using cDNA microarrays, this study examined a novel function of CD82 in CD34^+^/CD38^−^ AML cells, and found that CD82 positively regulates the expression of EZH2 via inactivation of p38 MPAK signaling, resulting in hypermethylation of the promoter regions of tumor suppressor genes, such as *PTEN* and *p16*.

## Material and Methods

### Isolation of leukemia cells from patients

Informed written consent was obtained from each subject in accordance with the Declaration of Helsinki. After obtaining written informed consent and Kochi University Institutional Review Board approval, AML patients (n = 19, Table 1) were minimally differentiated according to the World Health Organization (WHO) classification system of AML patient subtypes: minimally differentiated AML (n = 1, case 3), AML without maturation (n = 2, cases 1 and 14), AML with maturation (n = 3, cases 2 and 11), acute myelomonocytic leukemia (n = 3, cases 4, 10, and 13), AML with myelodysplasia changes (n = 5, cases 6, 7, 8, 12, and 16), and therapy-related AML (n = 3, cases 5, 9, and 15). CD34^+^/CD38^−^ cells and CD34^+^/CD38^+^ cells were purified by magnetic cell sorting utilizing MicroBead kits (Miltenyi Biotec GmbH, Bergisch Gladbach, Germany), as previously described [30].

**Table 1:** Patient’s characteristics.

Pt. #	WHO	cytogenetics	FLT3 mutation
1	AML without maturation	normal	ITD
2	AML with maturation	normal	WT
3	minimally differentiated AML	normal	WT
4	Acute myelomonocytic leukemia	normal	WT
5	Therapy-related AML	complex	WT
6	AML with myelodysplasia changes	del(7q)(q11.2)	NA
7	AML with myelodysplasia changes	normal	WT
8	AML with myelodysplasia changes	-7	WT
9	therapy-related AML	46XY	WT
10	Acute myelomonocytic leukemia	complex	WT
11	AML with myelodysplasia changes	t(11;19)(q23;p15.1)	WT
12	AML with myelodysplasia changes	-7	WT
13	Acute myelomonocytic leukemia	complex	NA
14	AML without maturation	NA	NA
15	Therapy-related AML	t(10;11)(p12;q23)	NA
16	AML with myelodysplasia changes	11q23	WT
17	AML with maturation	t(1;22)(q44;q12)	ITD
18	AML with maturation	t(11;13)(p13;q12)	NA
19	AML with myelodysplasia changes	complex	NA

Pt, patient; F, female; WHO, World Health Organization (leukemia classification); FLT3, fms-like tyrosine kinase 3; WT, wild-type; ITD, internal tandem duplication. NA, not assessed.

### Cells

Chronic eosinophilic leukemia (CEL) EOL-1 cells were obtained from the RIKEN BRC Cell Bank (Tsukuba, Japan). The establishment of the imatinib-resistant EOL-1R cell line has been described elsewhere [20]. The characteristics of the CEL EOL-1R cells are similar to those of LSCs; a high proportion of EOL-1R cells were arrested in the G0/G1 phase of the cell cycle in parallel with upregulation of p53 and p21^*waf1*^ [30]. EOL-1R cells overexpressed CD82 (96%) more frequently than EOL-1 cells (47%) [30]. The MOLM13 line of AMLM5a cells with FLT3/ITD was kindly provided by Dr. Yoshinobu Matsuo (Fujisaki Cell Center, Okayama, Japan) [32].

### Drugs

The p38 inhibitor, SB203580, was purchased from LC Laboratories (Woburn, MA USA). The EZH2 inhibitor, EZH2i (patent# A01N 43/38), was a kind gift from Constellation Pharmaceuticals (Cambridge, MA, USA). The EZH2 inhibitor, 3-Deazaneplanocin A (DZNep) was purchased from Sigma-Aldrich (St. Louis, MO, USA).

### Isolation of CD34^+^/CD38^-^/CD82^-^ and CD34^+^/CD38^-^/CD82^+^ AML cells and RT-PCR

Leukemia cells were stained with fluorescent-labeled antibodies fluorescein isothiocyanate (FITC)-anti-CD34 (Beckman Coulter, CA, USA), allophycocyanin (APC)-anti-CD38 (BioLegend, San Diego, USA), and phycoerythrin (PE)-anti CD82 (BioLegend) and separated by cell sorting using a FACS Aria II instrument (BD Biosciences, Heidelberg, Germany). RNA was extracted using the CellAmp Direct RNA Prep Kit for RT-PCR (Takara, Shiga, Japan) and reverse transcription was performed using the One Step PrimeScript RT-PCR Kit (Takara, Shiga, Japan). Amplification was conducted using a StepOne plus system (Life Technology, CA USA) at the following conditions: 42°C for 5 min, 95°C for 10 s, and 40 cycles at 95°C for 5 s, 55°C for 30 s, and 72°C for 1 min. The *18S* gene was used as internal control. PCR primers are listed in Table 2.

**Table 2:** PCR primers used in this study.

Gene	Direction	Primer
EZH2 v1	Forward	5’-TTCATGCAACACCCAACACT-3’
	Reverse	5’-GGGCCTGCTACTGTTATTGG-3’
CD82	Forward	5’-GGCGGGATGGGCTCAGCCTG-3’
	Reverse	5’-TCAGTACTTGGGGACCTTGC-3’
PTEN	Forward	5’-ACCAGGACCAGAGGAAACCT-3’
	Reverse	5’-GCTAGCCTCTGGATTTGACG-3’
p16	Forward	5’-GGTGCGGGCGCTGCTGGA-3’
	Reverse	5’-AGCACCACCAGCGTGTCC-3’
18S	Forward	5’-AAACGGCTACCACATCCAAG-3’
	Reverse	5’-CCTCCAATGGATCCTCGTTA-3’

### Extraction of RNA

Total RNA was extracted from tissues using a single-step Trizol RNA extraction kit (Invitrogen, California, CA, USA) according to the manufacturer’s instructions, then concentrated by isopropanol precipitation, and column-purified using a QIAGEN RNeasy Mini Kit (Cat No. 74104). The RNA was quantified using a Nanodrop ND-1000 spectrophotometer (Thermo Scientific, MA, USA). RNA quality was evaluated using Agilent 2100 Bioanalyzer expert software. RNA samples with a 2100 Bioanalyzer RNA integrity number ≥6.0 were considered of sufficient quality for use in gene expression profiling experiments.

### Microarray hybridization

Two hundred and fifty nanograms of each cDNA sample was used as template for Cy3 and Cy5 labeling, which was performed according to the undisclosed protocol of Miltenyi Biotec. Corresponding Cy3- and Cy5-labeled cDNAs were combined and hybridized overnight (17 h, 65°C) to an Agilent Whole Human Genome Oligo Microarray 8×60 K (Agilent Technologies, CA, USA) using the manufacturer’s recommended hybridization chamber and oven. In general, control samples were labeled with Cy3 and experimental samples were labeled with Cy5. The microarrays were washed once with Agilent Gene Expression Wash Buffer 1 for 1 min at room temperature, once with preheated Agilent Gene Expression Wash Buffer 2 for 1 min at 37°C, and once with acetonitrile. The washed hybridized microarrays were scanned using an Agilent DNA microarray scanner. The resulting images were analyzed using the Rosetta Resolver gene expression data analysis system (Rosetta Biosoftware, UK). The microarray data are available in National Center for Biotechnology Information (NCBI) Gene Expression Omnibus (GEO) under accession number GSE64527.

### Western blot analysis

Western blot analysis was performed as described previously [26] using the following antibodies: anti-p-p38 MAPK (#4511, Cell Signaling Technology, Beverly, MA), anti-p38 MAPK (#8690, Cell Signaling Technology), anti-EZH2 (#5246, Cell Signaling Technology), anti-CD82 (sc-17752, Santa Cruz Biotechnology, Santa Cruz, CA), and anti-GAPDH (ab9413, Abcam, Cambridge, UK).

### Immunoprecipitation

Whole proteins were extracted and immunoprecipitated using an anti-EZH2 antibody (#5246, Cell Signaling Technology). The precipitated samples were then subjected to western blot analysis. The membrane was sequentially probed with anti-phospho-threonine (#9381, Cell Signaling Technology) and anti-EZH2 (Cell Signaling Technology) antibodies.

### Methylation analysis

EOL-1 cells were transfected with either the control or *CD82*-expression vector for 48 h. EOL-1R and MOLM13 cells were pretreated with either IgG or CD82 mAb for 1 h on ice and then incubated for 48 h at 37°C, after which DNA was extracted from these cells.

Three hundred nanograms of DNA isolated from these cells were treated with bisulfite using an EZ DNA Methylation kit (Zymo Research, Orange, CA, USA) according to the supplier’s protocol. The methylation-specific primer sequences for the *PTEN* and *p16* promoters were as follows: forward, 5’-TTCGTTCGTCGTCGTCGTATTT-3’ and 5’-TTATTAGAGGGTGGGGCGGATCGC-3’, respectively; reverse, 5’-GCCGCTTAACTCTAAACCGCAACCG-3’ and 5’-GACCCCGAACCGCGACCGTAA-3’, respectively. The non-methylation-specific primer sequences for the *PTEN* and *p16* promoters were as follows: forward, 5’-GTGTTGGTGGAGGTAGTTGTTT-3’ and 5’-TTATTAGAGGGTGGGGTGGATTGT-3’, respectively; reverse, 5’-GTGTTGGTGGAGGTAGTTGTTT-3’ and 5’-CAACCCCAAACCACAACCATAA-3’, respectively. Amplification was carried out in a Mycycler thermal cycler (Bio-Rad, Tokyo, Japan). PCR conditions were as follows: 94°C initial activation for 1 min followed by 30 cycles of 98°C for 10 s, 59°C for 15 s, and 68°C for 30 s.

### RNA isolation and real-time reverse-transcription polymerase chain reaction (RT-PCR)

RNA isolation and real-time RT-PCR were carried out as described previously [33]. *GAPDH* expression was measured for normalization. The primers used for PCR are shown in Table 1.

### Small interfering (si)RNAs and transfection

Control siRNA and siRNAs against *CD82* were purchased from Santa Cruz Biotechnology and Sigma (Deisenhofen, Germany), respectively. These siRNAs were transfected into leukemia cells using a Nucleofector Kit V (program U-001 and X-001), as previously described [30]. CD82 siRNA1; CTGTATCAAAGTCACCAAA, CD82 siRNA2; CTGAGGACTGGCCTGTGTA.

### Production of CD82 shRNA, lentiviral vectors, and transduction of cells

Lentiviral CD82 shRNA particles coexpressing GFP were generated using the ViraPower packaging system (Invitrogen) as described previously [30]. Lentiviral CD82 shRNA particles and polybrene (10 μg/ml) were added to leukemia cells (5 × 10^4^) being cultured in serum free Iscove’s modified Dulbecco’s medium (IMDM) (500 μl). On the following day, 1 ml of methylcellulose medium H4034 (StemCell Technologies, Vancouver, BC, Canada) was added to this culture and cells were incubated for 7 days, which yielded nearly a 70% transduction efficiency [30]. CD82 shRNA1; CTCTTCAACTTGATCTTCT, CD82 shRNA2; TGACTTCGCAGGAACAGGG.

### CD82 lentiviral vectors

CD82 expressing lentiviral particles were generated using the ViraPower packaging system (Invitrogen), as described previously [30].

### Anti-CD82 monoclonal antibody (CD82 mAb)

The binding of human CD82 mAb (Santa Cruz Biotechnology) to the surface of EOL-1R cells was confirmed using an Olympus FV1000-D microscope (data not shown).

### Chromatin immunoprecipitation (ChIP) assay

ChIP assays were performed using a ChIP assay kit (Cell Signaling Technology) according to the manufacturer’s instructions. An anti-*EZH2* (Cell Signaling Technology) or-H3K27me3 (#9733, Cell Signaling Technology) antibody was used for immunoprecipitation. DNA samples were purified and used for real-time PCR, which was performed using the following set of specific primers: *p16*, forward 5’-ACCCCGATTCAATTTGGCAG-3’ and reverse 5’-AAAAAGAAATCCGCCCCCG3-3’ [34]; *PTEN*, forward 5’-CGGGCGGTGATGTGGC-3’ and reverse 5’-GCCTCACAGCGGCTCAACTCT-3’. The PCR conditions for all genes were as follows: 95°C initial activation for 10 min followed by 40 cycles of 95°C for 15 s and 60°C for 60 s. Finally, fluorescence was determined for 20 s at the melting temperature of the product. All steps were performed using a StepOnePlus real-time PCR system (Applied Biosystems).

### FACS

The level of H3K27me3 in CD34^+^/CD38^−^ AML cells was assessed by flow cytometry using an anti-trimethyl-histone H3 (Lys27) (C36B11) antibody (#9733, Cell Signaling Technology).

### Statistical analysis

The Student’s *t*-test was carried out to assess the difference between two groups. SPSS software (Version 11.03; SPSS, Tokyo, Japan) was utilized for statistical analysis. Differences were considered significant when the *P*-value was <0.05 and highly significant when the *P*-value was <0.01.

## Results

### Gene expression profiles in leukemia cells following short hairpin (sh)RNA-mediated downregulation of *CD82* gene expression

**Fig 1:**
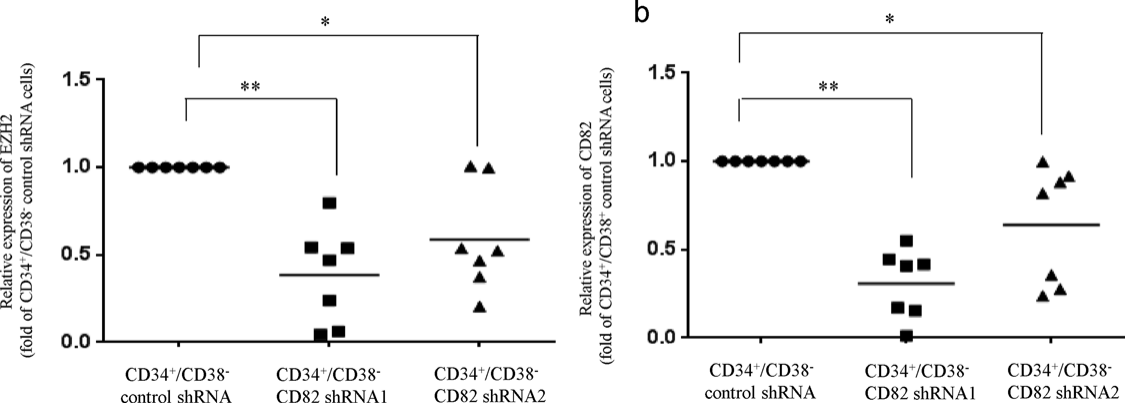
Effect of CD82 on expression of EZH2 in CD34^+^/CD38^−^ AML cells. **Real-time RT-PCR.** (a, b) CD34^**+**^/CD38^**−**^ AML cells (n = 7) transduced with CD82 shRNA (CD82 shRNA1 and CD82 shRNA2) lentiviral particles were collected and mRNA was extracted. cDNA was synthesized and subjected to real-time RT-PCR to determine the levels of EZH2 and CD82 expression. Each dot represents the level of EZH2 expression for an individual experiment, and the mean is indicated by the line. ** P < 0.01; * P < 0.05. The levels of EZH2 and CD82 in CD34^**+**^/CD38^**-**^ cells and their CD34^**+**^/CD38^**+**^ counterparts. Real time RT-PCR. (c) RNA was extracted from CD34^**+**^/CD38^**-**^ cells and their CD34^**+**^/CD38^**+**^ counterparts isolated from AML patients (n = 10). cDNAs were synthesized and subjected to real-time RT-PCR to measure the levels of EZH2. Each dot represents the levels of EZH2 for an individual experiment and the mean is indicated by the line. ** P < 0.01; * P < 0.05. (d) The correlation trend between EZH2 and CD82 in CD34^**+**^/CD38^**-**^ cells. Real time RT-PCR. (e) CD34^**+**^/CD38^**−**^/CD82^**+**^ and CD34^**+**^/CD38^**−**^/CD82^**−**^ AML (n = 12) cells were sorted and mRNA was extracted. cDNAs were synthesized and subjected to real-time RT-PCR to measure the levels of EZH2. Each dot represents the levels of EZH2 for an individual experiment and the mean is indicated by the line. Real time RT-PCR. (f) CD34^**+**^/CD38^**+**^ AML cells (n = 6) transduced with CD82-expressing lentiviral particles were collected and mRNA was extracted. cDNA was synthesized and subjected to real-time RT-PCR to determine the level of CD82 and EZH2 expression. Each dot represents these levels for an individual experiment, and the mean is indicated by the line. ** P < 0.01; * P < 0.05.

To explore the function of CD82 in CD34^+^/CD38^−^ AML cells, CD34^+^/CD38^-^ cells isolated from AML patients (n = 3, cases #1–3) were transduced with CD82-specific shRNA and their gene expression profiles were compared to the cells transduced with control shRNA by microarray analysis (GSE64527). Three-fold increases or decreases in expression for more than 2 AML cases were considered significant. We identified a total of 995 genes that were differentially expressed between the CD82-depleted CD34^+^/CD38^−^ AML cells and the control cells (data not shown). Notably, a series of epigenetic regulator genes, including those encoding the polycomb group protein EZH2, DNMT3A, and histone deacetylase 6 were downregulated (S1 Table). In contrast, expression of the CDKN2A (p16) and CDKN1B genes, which are negative regulators of the cell cycle, were upregulated in the CD82-depleted CD34^+^/CD38^−^ AML cells (S1 Table). To validate the microarray results, we performed real-time RT-PCR, which showed that the EZH2 expression level decreased 0.4-fold in CD34^+^/CD38^−^ AML cells following shRNA-mediated downregulation of CD82 expression (n = 7; Fig 1A and 1B) [30, 31]. Moreover, we found that the levels of EZH2 and CD82 were 4-fold and 6-fold higher, respectively, in CD34^+^/CD38^−^ AML cells compared with CD34^+^/CD38^+^ cells (n = 10; Fig 1C). Further, we utilized these results to examine the correlation between CD82 and EZH2. Aanalyses of CD34^+^/CD38^−^ AML cells (n = 10) showed a positive correlation (r = 0.33) between the mRNA expression levels of CD82 and EZH2 (Fig 1D). These observations led us to hypothesize that CD82 regulates the expression of EZH2 in CD34^+^/CD38^−^ AML cells. For this purpose, we employed real-time RT-PCR to compare the levels of EZH2 in CD34^+^/CD38^−^/CD82^+^AML cells and their CD34^+^/CD38^−^/CD82^−^ counterparts isolated from AML patients (n = 12; Fig 1E). The CD34^+^/CD38^−^/CD82^+^ and CD34^+^/CD38^−^/CD82^−^ cells were sorted (S1A and S1B Fig), and, as expected, the level of EZH2 expression was 24-fold greater in CD34^+^/CD38^−^/CD82^+^AML cells compared with the CD34^+^/CD38^−^/CD82^−^ group (n = 12; Fig 1E; P = 0.065). In contrast, the level of KRAS expression was not affected, excluding the possibility of off-target effects of CD82 (S1C Fig). Additional experiments found that transduction of CD82-expressing lentiviral particles into CD34^+^/CD38^+^ AML cells increased EZH2 levels by a factor of 3.6 (n = 6, Fig 1F), which was in agreement with the observed increase in CD82 expression (Fig 1F) [30, 31], and further supports our hypothesis that CD82 regulates the expression of EZH2 in CD34^+^/CD38^−^ AML cells.

### CD82 increases the level of EZH2 in leukemia cells via inactivation of p38 MAPK signaling

**Fig 2:**
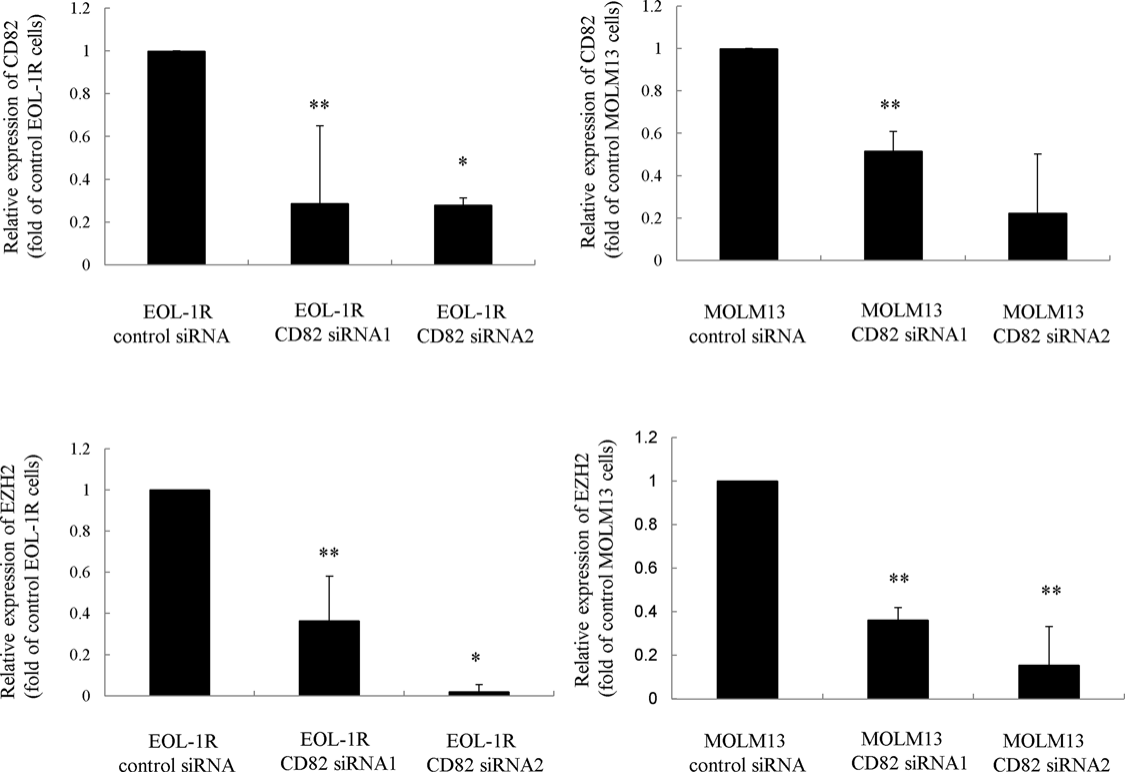
Effect of p38 MAPK on expression of EZH2 in leukemia cells. **Real time RT-PCR.** (a) EOL-1R and MOLM13 cells were transiently transfected with either control or CD82 siRNAs. After 24 h, cells were collected and mRNAs were extracted. Real time RT-PCR. (b) EOL-1 cells transduced with CD82-expressing lentiviral particles were collected and mRNA was extracted. cDNA was synthesized and subjected to real time RT-PCR to measure the levels of CD82 and EZH2 expression. Results represent the mean ± SD of three experiments performed in duplicate. ** P < 0.01; * P < 0.05. Western blot analysis. (c) EOL-1 cells were treated with the p38 inhibitor, SB203580 (10 μM), for 3 h before the cells were harvested and subjected to western blot analysis to determine the relative levels of the indicated proteins. Each lane was loaded with 30 μg of whole-protein lysate. Band intensities were quantified using ImageJ software (Wayne Rasband, NIH). These experiments were repeated three times. Real time RT-PCR. (d) EOL-1 cells were treated with SB203580 (10 μM). After 3 h, cells were collected and mRNA was extracted. cDNA was synthesized and subjected to real time RT-PCR to measure the levels of CD82 and EZH2 expression. Results represent the mean ± SD of three experiments performed in duplicate. ** P < 0.01; * P < 0.05. Immunoprecipitation. (e) Cells were harvested and proteins were extracted. EZH2 was immunoprecipitated and subjected to western blot analysis. The membrane was probed sequentially with anti-phospho-threonine (P-Thr-Polyclonal) (top) and anti-EZH2 antibodies (bottom). These experiments were repeated three times. p-Threonine; phosphor-Threonine. Effect of CD82 on expression of EZH2 in leukemia cells. Western blot analysis. (f) EOL-1 cells were transfected with either the CD82-expression vector or the control vector for 48 h. EOL-1R and MOLM13 cells were pretreated with either IgG or CD82 mAb for 1 h on ice and then incubated for 24 h at 37°C, after which the cells were harvested and subjected to western blot analysis to determine the relative levels of the indicated proteins. Each lane was loaded with 30 μg of whole-protein lysate. Band intensities were quantified using ImageJ software (Wayne Rasband, NIH). These experiments were repeated three times. Immunoprecipitation. (g) Cells were harvested and proteins were extracted. EZH2 was immunoprecipitated and subjected to western blot analysis. The membrane was probed sequentially with anti-phospho-threonine (top) and anti-EZH2 antibodies (bottom). These experiments were repeated three times. P-Threonine; phosphor-Threonine. Western blot analysis. (h) EOL-1R and MOLM13 cells were transiently transfected with either control or CD82 siRNAs. After 24 h, cells were treated with the p38 inhibitor, SB203580 (10 μM), for 3 h before the cells were harvested and subjected to western blot analysis to determine the relative levels of the indicated proteins. Each lane was loaded with 30 μg of whole-protein lysate. Band intensities were quantified using ImageJ software (Wayne Rasband, NIH). These experiments were repeated three times.

To verify the molecular mechanism by which CD82 regulates the expression of EZH2, we examined CEL EOL-1R and AML MOLM13 cells. Downregulation of CD82 in EOL-1R and MOLM13 cells by siRNAs resulted in a decrease in EZH2 level (Fig 2A). In contrast, the levels of KRAS were not affected, excluding the possibility of off-target effects of the siRNA (data not shown). As expected, forced expression of CD8*2* in EOL-1 cells increased the level of EZH2 by a factor of 4 (Fig 2B and S2 Fig). In addition, expression of EZH2 was induced in response to inhibition of MAPK pathway in human breast cancer cells [35]. We found that a 3-hour exposure of EOL-1 cells to the selective p38 inhibitor, SB203580 (10 μM), resulted in a decrease in the level of p-p38 and an increase in the levels of both phosphorylation at threonine residues and total EZH2 (Fig 2C–2E), suggesting that p38 MAPK signaling negatively regulates the expression of EZH2. We then examined whether CD82 inactivates p38 in leukemia cells, and found that forced expression of CD82 in EOL-1 cells results in a 5-fold decrease in the level of p-p38 and increases in both phosphorylation at the threonine residues and total EZH2 (Fig 2F and 2G). In addition, forced expression of CD82 increased the level of H3K27me3 in these cells (Fig 2F). However, an antibody-mediated blockade of CD82 (1 μg/mL, 24 h) in EOL-1R or MOLM13 cells resulted in approximately a 2-fold increase in the level of p-p38 and a 2-fold decrease in the both phosphorylation at the threonine residues and total EZH2 (Fig 2F and 2G). Moreover, we found that p38 inhibitor SB203580 enhanced the expression of EZH2 in CD82-downregulated EOL-1R and MOLM13 cells (Fig 2H).

### Inhibition of CD82 decreases the amount of EZH2 bound to the *PTEN* promoter and interferes with trimethylation of H3K27 in this region in leukemia cells

**Fig 3:**
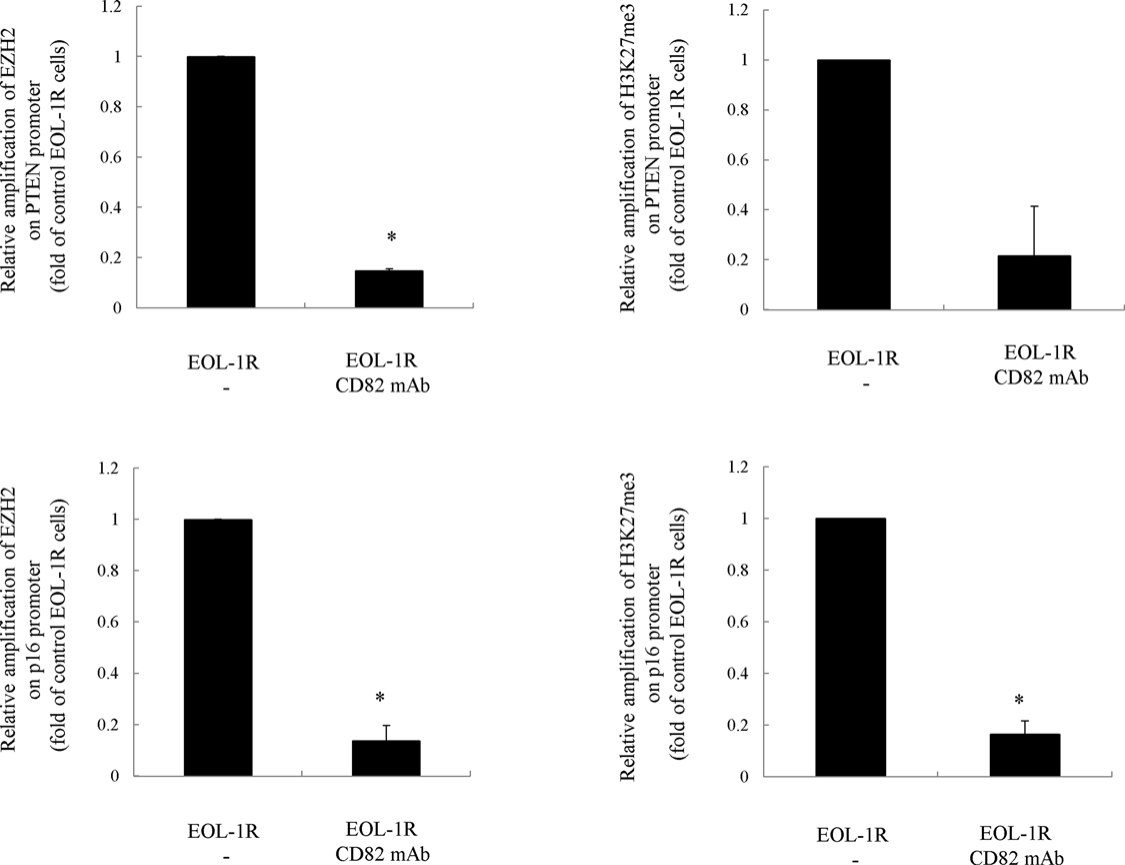
Effect of CD82 inhibition in leukemia cells on both the amount of *PTEN* and *p16* promoter-bound EZH2 and the level of H3K27 trimethylation in this promoter region. **ChIP assay.** (a) EOL-1R and (b) MOLM13 cells were pretreated with either IgG or anti-CD82 for 48 h. The cells were then harvested and subjected to chromatin immunoprecipitation followed by real-time RT-PCR. The amplified sequences of the *PTEN* and *p16* promoter were normalized to those of the input (the cross-linked DNA/protein complexes, which were not immunoprecipitated with the anti-EZH2 or-H3K27me3 antibodies). These experiments were repeated three times.

To determine whether CD82 expression in leukemia cells affects the amount of *PTEN* promoter-bound EZH2 or the trimethylation status of H3K27 in this region, we performed ChIP assays using anti-EZH2 or-H3K27me3 antibodies in EOL-1R and MOLM13 cells (Fig 3A and 3B). Antibody-mediated inhibition of CD82 in these cells decreased the amount of EZH2 bound to the *PTEN* or *p16* promoter and inhibited trimethylation of H3K27 in this region (Fig 3A and 3B).

### The effect of CD82 inhibition on DNA hypermethylation

We previously showed that DNMT3A forms a complex with EZH2, which then facilitates the binding of these proteins to the *PTEN* promoter and induces DNA hypermethylation in this region [19]. In addition, enriched EZH2 at the p16 transcription-start-site maintains H3K27 methylation status and suppresses the expression of p16 in mixed lineage leukemia fusion leukemia [36]. We therefore hypothesized that inhibition of CD82, which results in EZH2 downregulation, would affect epigenetic modifications on the *PTEN* and *p16* gene, such as DNA hypermethylation.

**Fig 4:**
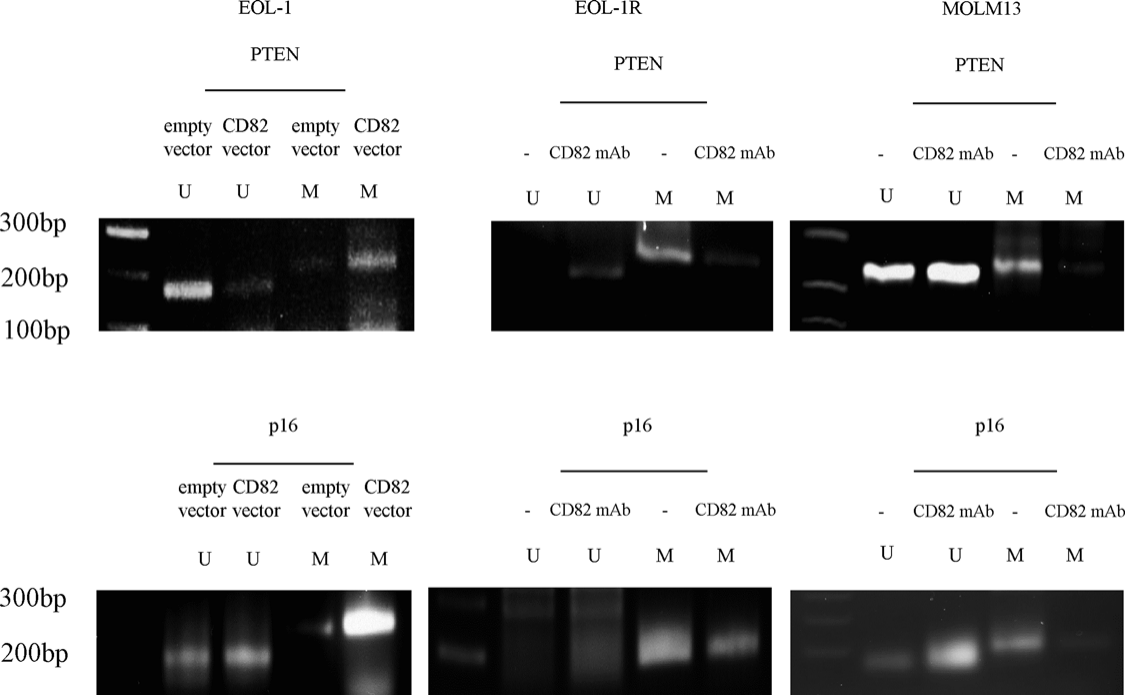
Methylation-specific PCR. (a) EOL-1 cells were transfected with either the control or *CD82*-expression vector. EOL-1R and MOLM13 cells were pretreated with either IgG or anti-CD82 monoclonal antibody (CD82 mAb). DNA was extracted from these cells and methylated CpG was processed using an EZ DNA Methylation Kit. The recovered DNA was amplified by PCR on methylation of the *PTEN* and *p16* promoter. The lane at extreme left in each panel is a size marker. M, methylated of the gene promoter; U, unmethylated of the gene promoter. The experiments were repeated three times. Real-time RT-PCR. (b) RNA was extracted and cDNAs were synthesized and subjected to real-time RT-PCR to measure the level of *PTEN* and *p16*. Results represent the mean ± SD of three experiments performed in duplicate. ** *P* < 0.01; * *P* < 0.05. Western blot analysis. (c) EOL-1 cells were transfected with either the control or *CD82*-expression vector for 72 h. EOL-1R and MOLM13 cells were pretreated with either IgG or anti-CD82 for 1 h on ice, and then incubated for 72 h at 37°C before the cells were harvested and subjected to western blot analysis to determine the relative levels of the indicated proteins. Each lane was loaded with 30 μg of whole-protein lysate. Band intensities were quantified using ImageJ software (Wayne Rasband, NIH). Real-time RT-PCR. (d) CD34^**+**^/CD38^**−**^/CD82^**+**^ and CD34^**+**^/CD38^**−**^/CD82^**−**^ AML (n = 5) cells were sorted and mRNA was extracted. cDNA was synthesized and subjected to real time RT-PCR to measure the levels of the indicated expression. Each dot represents the level for an individual experiment, and the mean is indicated by the line. ** *P* < 0.01; * *P* < 0.05. Real-time RT-PCR. (e) EOL-1R and MOLM13 cells were treated with EZH2 inhibitor (DZNep) (1 μM). After 48 h, cells were collected and mRNA was extracted. cDNA was synthesized and subjected to real time RT-PCR to measure the levels of MMP9 expression. Results represent the mean ± SD of three experiments performed in duplicate. * P < 0.05.

To test this hypothesis, we examined the methylation status of the *PTEN* and *p16* promoter region in leukemia cells utilizing methylation-specific PCR. EOL-1 cells were transfected with either the control or *CD82*-expression vector. EOL-1R and MOLM13 cells were pretreated with either IgG or CD82 mAb. Interestingly, hypermethylation of the *PTEN* or *p16* promoter region in EOL-1R and MOLM13 cells decreased after treatment with CD82 mAb for 48 h (Fig 4A); this was accompanied by an increase in these gene transcript levels. In contrast, when EOL-1 cells were transfected with a *CD82*-expression vector, expression of PTEN was downregulated at both the mRNA and protein levels (Fig 4B and 4C). Moreover, we found that the levels of both PTEN and p16 in CD34^+^/CD38^−^/CD82^+^ AML cells were lower than they were in CD34^+^/CD38^−^/CD82^−^ AML cells (Fig 4D). These observations suggest that CD82 downregulates the expression of the *PTEN* and *p16* gene in leukemia cells via epigenetic mechanisms.

### The effect of EZH2 on MMP9 in leukemia cells

CD82 negatively regulated MMP9 and modulated adhesion to BM in AML cells [24, 25]. We next examined whether CD82-induced EZH2 repressed the levels of MMP9 in leukemia cells. As expected, EZH2 inhibitor (DZNep) upregulated the levels of MMP9 in EOL-1R and MOLM13 cells (Fig 4E).

## Discussion

The present study found that CD82 positively regulates both the expression and phosphorylation of EZH2 in leukemia cells via inactivation of p38 MAPK signaling (Figs 1 and 2). Moreover, the p38 inhibitor, SB203580, induced EZH2 expression in CD82-downregulated leukemia cells (Fig 2), suggesting that p38 directly downregulated the expression of EZH2. Phosphorylation of EZH2 plays an essential role in promoting H3K27me3 and silencing chromatin targets, such as Hox loci [37]. This led us to hypothesize that CD82 may regulate DNA hypermethylation via phosphorylation of EZH2. Indeed, our results confirm that antibody-mediated inhibition of CD82 in leukemia cells interferes with DNA hypermethylation of the *PTEN* promoter region, and is associated with a decrease in both the level of H3K27me3 and the amount of EZH2 bound to the promoter. These changes culminate in an increase in the expression of the PTEN tumor suppressor (Figs 3 and 4). These results suggest that CD82 mediates epigenetic silencing of PTEN via upregulation of the expression and phosphorylation of EZH2. CD82 overexpression or downregulation did not affect morphology of AML cells in short time culture (data not shown).

EZH2 suppresses differentiation programs in leukemic stem cells, thereby augmenting their leukemogenic activity [38]. We confirmed that exposure of CD34^+^/CD38^−^ AML cells to the EZH2 inhibitor EZH2i increased the levels of both PTEN and p16 in parallel with suppression of H3K27me3 (S3 Fig). Thus, EZH2 might inhibit the expression of tumor suppression genes in response to trimethylation of H3K27 in the *PTEN* promoter region and augment the self-renewal capability of AML cells.

*PTEN* is commonly deleted or otherwise inactivated in diverse forms of cancer, including hematopoietic malignancies [39, 40]. Deletion of *PTEN* in HSCs leads to a myeloproliferative disease that progresses to AML and acute lymphoblastic leukemia (ALL) [41]. In contrast, forced expression of *PTEN* suppresses development of CML and B-cell ALL (B-ALL) in mice [42]. These observations suggest that PTEN plays an important role in both leukemogenesis and the maintenance of stem cell activity. Hence, downregulation of PTEN expression by the CD82/EZH2-axis may contribute to leukemogenesis.

SB20358 promotes expansion and engraftment of human umbilical cord blood (hUCB) CD133^+^ cells in immunodeficient mice. SB203580 also inhibits senescence and apoptosis in hUCB HSCs, and dramatically enhances the stem cell activity of hUCB HSCs [43]. In contrast, p38 MAPK activation and upregulation of p16 induce HSC exhaustion/senescence [44–47]. This is in agreement with other reports, which claim that p16 plays a central role in the induction and maintenance of cellular senescence [48]. In our study, CD82 inactivated p38 MAPK and downregulated expression of p16 in leukemia cells (Fig 4B–4D), suggesting that CD82 might inhibit senescence in CD34^+^/CD38^−^ AML cells via inactivation of p38 MAPK signaling and suppression of p16 expression. Other study also showed that inhibition of the p38 MAPK signal pathway blocks the induction of p16 expression and cellular senescence in endothelial progenitor cells [46].

Recurrent gain-of-function mutations targeting EZH2^Y641^ are associated with hyperactivation of H3K27me3 in diffuse large B cell lymphoma cells [13, 49–51]. Moreover, EZH2^Y641F^ can collaborate with Myc to accelerate lymphomagenesis *in vivo*, suggesting a cooperative role of EZH2 mutations in oncogenesis [52]. On the other hand, other investigators showed that gain of function mutation EZH2^Y641^ was not found in AML patients [17]. Loss of function EZH2 mutations were identified in only 1.7% of de novo AML and this type of mutations were observed more frequently in cases with -7/del(7q) than in cases without -7/del(7q). Missense, nonsense, and frameshift mutations were found in de novo AML and involved in exons 2, 4, 9, 12–14, 17–20. No significant difference in overall survival was noted between AML patients with and without EZH2 mutation [17]. We also examined EZH2 mutation in AML patients with -7/del(7q) (case #s 3 and 8) and without -7/del(7q) (case # 12). As a result, EZH2 mutations (exons 2, 4, 9, 12–15, and 17–20) were not found in these cases (data not shown). Activated EZH2, rather than loss of function EZH2 may play a role in leukemogenesis in AML.

In this study, EZH2i had a modest inhibitory effect on colony formation by CD34^+^/CD38^−^ AML cells (S3 Fig). However, many other factors support the survival of leukemia cells, including STAT5, which plays a crucial role in maintaining leukemic stem cells in AML [53]. This may account for the fact that inhibition of EZH2 did not completely block colony formation by CD34^+^/CD38^−^AML cells in our model.

**Fig 5:**
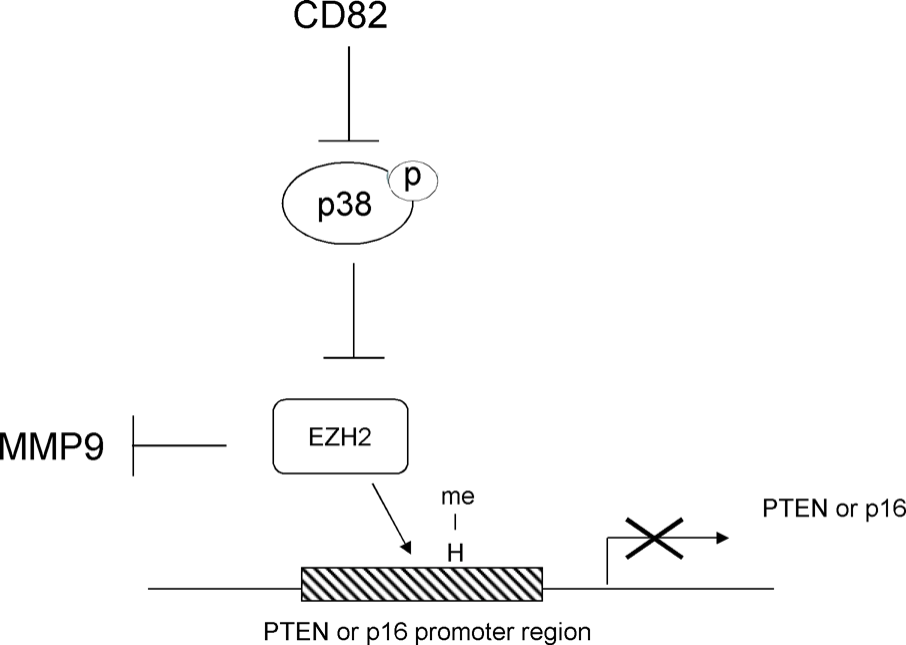
*CD82* positively regulates the expression of *EZH2* via inactivation of p38 MPAK signaling.

We previously reported that CD82 negatively regulated MMP9 and modulated adhesion to BM [30]. Notably, this study showed that EZH2 inhibitor (DZNep) increased the levels of MMP9 in leukemia cells (Fig 4). Other study suggested that epigenetic repression of MMP9 by EZH2 maintained the integrity of the developing vasculature in endothelial cells [54]. Moreover, SB203580 downregulates the expression of MMP9 and MT1-MMP/MMP-14 in fibronectine-stimulated macrophages, and inhibits TNF-α-stimulated leukocyte migration across fibronectine [55]. Theoretically, these puzzle pieces may come together in a pathway where CD82 negatively regulates p38 MAPK signaling and increase the expression of EZH2, which decreases MMP9 expressions in AML cells (Fig 5). This signal pathway may modulate adhesion of leukemia cells to BM niche.

We previously found that CD82 directly interacted with integrin a4 [30]. CD82/integrin a4b1 (VLA4) signal pathway may associate with inactivation of p38 MAPK. Blockade of CD82 by an antibody may disrupt interaction between CD82 andVLA4, resulting in activation of p38 MAPK.

MMPs cleave integrin b4 and b1 in cultured human corneal epithelial cells and mouse epidermal keratinocytes, suggesting that activation of MMP9 by down-regulation of CD82 may disrupt interaction between VLA4 and vascular cellular adhesion molecule-1 on cell surface of stromal cells. The interaction between vascular cellular adhesion molecule-1 and VLA-4 played an integral role in the activation of NF-κB in the stromal and tumor cell compartments [56]. Thus, blockade of CD82 by an antibody could inactivate NF-κB.

Taken together, our results suggest that CD82 regulates the expression of EZH2 in leukemia cells via p38 MAPK signaling, resulting in both H3K27me3 and methylation in the *PTEN* promoter region, thereby resulting in repression of *PTEN* transcription. Thus, CD82 may play a role as an epigenetic regulator in CD34^+^/CD38^−^ AML cells.

## Supporting Information

S1 Fig Sorting. (a) CD34^+^/CD38^-^/CD82^+^cells and their CD34^+^/CD38^-^/CD82^-^ counterparts were sorted from AML cells. (b) CD82 expression in CD34^+^/CD38^-^/CD82^+^cells. Real time RT-PCR. (c) RNA was extracted from CD34^+^/CD38^-^/CD82^+^cells and their CD34^+^/CD38^-^/CD82^-^ counterparts isolated from AML patients (n = 12). cDNAs were synthesized and subjected to real-time RT-PCR to measure the levels of KRAS. Each dot represents the levels of KRAS for an individual experiment and the mean is indicated by the line.
(TIF)

S2 Fig FACS. CD82 expression in EOL-1 cells transduced CD82 vector or empty vector.
(TIF)

S3 Fig The effect of EZH2 on tumor suppressor genes in leukemia cells. Real time RT-PCR. (a) MOLM13 cells were treated with EZH2i (1 μM). cDNAs were synthesized and subjected to real time RT-PCR to measure the levels of PTEN and p16. Results represent the mean ± SD of three experiments performed in duplicate. * *P* < 0.05. Western blot analysis. (b) MOLM13 cells were treated with EZH2i (1 μM). These cells were harvested, and subjected to Western blot analysis to monitor the levels of the indicated proteins. Each lane was loaded with 30 μg of whole protein lysate. Band intensities were quantified using ImageJ software (Wayne Rasband, NIH). The lane at extreme left in each panel is a size marker. Colony forming assay. (c) CD34^+^/CD38^-^ (n = 6) were treated with EZH2 inhibitor EZH2i (400, 800 nM). These cells were cultured in methylcellulose medium. After 16 days, colonies were counted. ** *P* <0.01. FACS. (d) CD34^+^/CD38^-^ AML cells (n = 5) were treated with EZH2i (800 nM, 5 days). Cells were harvested, fixed, incubated with anti-Tri-Methyl-Histone H3 (Lys27) (H3K27me3) antibody, and measured the levels of H3K27me3 using flow cytometry. H3K27me3; trimethylate histone H3 at lysine 27. Real time RT-PCR. (e) CD34^+^/CD38^-^ AML cells (n = 6) were treated with EZH2i (800 nM). cDNAs were synthesized and subjected to real time RT-PCR to measure the levels of PTEN and p16. Each dot represents these levels for an individual experiment and the mean is indicated by the line.
(TIF)

S1 Table Gene expression profiles in CD34^+^/CD38^-^ AML cells transduced by either control or CD82 shRNA. Three-fold increases or decreases in expression for more than 2 AML cases were considered significant. We identified a total of 995 genes that were differentially expressed between the CD82-depleted CD34^+^/CD38^−^ AML cells and the control cells.
(DOC)
