# Supplementary material for: Tetraspanin Family Member, CD82, Regulates Expression of EZH2 via Inactivation of p38 MAPK Signaling in Leukemia Cells
Source: PLoS One. 2015 May 8;10(5):e0125017. doi: 10.1371/journal.pone.0125017 (PMC4425466; doi:10.1371/journal.pone.0125017)
Supplement: S1 Table — Three-fold increases or decreases in expression for more than 2 AML cases were considered significant. We identified a total of 995 genes that were differentially expressed between the CD82-depleted CD34+/CD38− AML cells and the control cells. (DOC) [file pone.0125017.s004.doc]

**Supplemental Table 1. Gene expression profiles in CD34^+^/CD38^-^ AML cells transduced by either control or CD82 shRNA.**

|  |  |  | #1 | #2 | #3 |
| --- | --- | --- | --- | --- | --- |
| Sequence Code | Primary Sequence Name | Accession # | Fold Change | Fold Change | Fold Change |
| A_33_P3252196 | EZH2 | NM_004456 | -44.57607 | 1 | -45.41325 |
| A_23_P43490 | CDKN2A | NM_058197 | 1.5062 | 12.96202 | 12.6114 |
| A_24_P81841 | CDKN1B | NM_004064 | 1.1729 | 5.53818 | 2.14756 |
| A_33_P3329187 | DNMT1 | NM_001130823 | -6.25463 | 1 | 1.94225 |
| A_33_P3272330 | DNMT3A | NM_175629 | -56.06986 | -546.8819 | -24.12942 |
| A_23_P28953 | DNMT3B | NM_175850 | 7.59961 | 1.14255 | -1.41695 |
| A_23_P114656 | HDAC1 | NM_004964 | -1.44689 | 1.99649 | -1.95255 |
| A_23_P122304 | HDAC2 | NM_001527 | 6.69999 | 1.63066 | -1.16498 |
| A_33_P3309929 | HDAC3 | NM_003883 | -2.62188 | -2.5477 | 1.22811 |
| A_23_P210048 | HDAC4 | NM_006037 | 1.19633 | 3.42031 | 1.80017 |
| A_24_P125283 | HDAC5 | NM_001015053 | -5.61387 | 3.24317 | 2.41651 |
| A_33_P3320619 | HDAC6 | NM_006044 | -4.98028 | 1.34215 | -11.08783 |
| A_23_P2582 | HDAC7 | NM_015401 | 1.47598 | 1.43786 | -1.79928 |
| A_23_P84922 | HDAC8 | NM_018486 | 5.59716 | 1 | -1.27154 |
| A_24_P206317 | HDAC9 | NM_058176 | 3.65939 | -2.21 | 1.44946 |
| A_23_P368740 | HDAC10 | NM_032019 | 1.07591 | 3.19124 | 2.48681 |
| A_33_P3417944 | HDAC11 | NM_001136041 | -5.5797 | 1.55014 | 1.7356 |

Three-fold increases or decreases in expression for more than 2 AML cases were considered significant. We identified a total of 995 genes that were differentially expressed between the CD82-depleted CD34^+^/CD38^−^ AML cells and the control cells.
